# A case report of a severe neonatal systemic vasculitis on the first day of life

**DOI:** 10.1186/s12969-021-00618-x

**Published:** 2021-10-30

**Authors:** Stephanie Wong, Erkan Demirkaya, Roberta Berard

**Affiliations:** 1 British Columbia Children’s Hospital, 4480 Oak Street Ambulatory care building, Vancouver, British Columbia V6H 3V4 Canada; 2 University of British Columbia, Vancouver, Canada; 3 Children’s Hospital, London Health Sciences Centre, London, Ontario Canada; 4 Western University, London, Canada

**Keywords:** Vasculitis, Polyarthritis, Pregnancy, Neonate

## Abstract

**Background:**

Neonatal systemic vasculitis syndromes have been reported in infants born to mothers with systemic lupus erythematosus, Sjögren’s syndrome, Behҫet’s disease, cutaneous polyarteritis nodosa and anti-neutrophil cytoplasmic antibody-associated vasculitides. Here we report a novel association of a case of new-onset maternal seronegative inflammatory arthritis associated with a transient systemic vasculitis in a neonate.

**Case presentation:**

In the first 24 h of life, a preterm Caucasian baby boy was noted to have blue discoloration to all four extremities. Despite antibiotics, fresh frozen plasma and anticoagulation, the discoloration remained, particularly in the left index finger. This was associated with fever and a maximum C-reactive protein (CRP) of 148 mg/L. Intravenous immunoglobulin (IVIG) was given with short-term improvement. Initial echocardiogram showed enlarged coronary arteries with normalization on repeat 1 week later. Clinical signs and symptoms responded to high dose oral steroid administration. MRI angiography (MRA) of the body and heart showed tortuosity of arteries in the upper and lower extremities with gadolinium uptake, suggestive of vasculitis. Autoantibody profile negative. Genetic panel for hereditary autoinflammatory diseases was negative as was whole exome sequencing performed on the trio. The baby was weaned off steroids by 5 months of age. A small distal autoamputation of the left index finger occurred.

He was born to a 28-year-old woman who developed new onset severe symmetrical polyarthritis at 8 weeks gestation. This was presumed a reactive arthritis secondary to a dental infection. Infectious work up and autoantibodies were negative. She was treated with high dose prednisone for the remainder of her pregnancy.

The mother was weaned off prednisone, treated with hydroxychloroquine for 8 months post-partum and remains in remission. A repeat MRA done at 1 year old showed mild residual tortuosities of the arteries in the forearms. The remainder of the medium and large vessels were within normal limits with no gadolinium enhancement to suggest active disease. The child is now 4 years old with normal growth and development.

**Conclusion:**

This is a unique case of new-onset seronegative presumed reactive arthritis in a mother with the rare development of a successfully treated medium vessel vasculitis in an infant.

**Supplementary Information:**

The online version contains supplementary material available at 10.1186/s12969-021-00618-x.

## Background

During pregnancy many changes occur in the immune system to allow tolerance to the fetus. A level of immunosuppression is required as the fetus expresses paternally inherited alloantigens which requires the mother to create a sense of tolerance. One mechanism of tolerance suggested is the switch from Th1 cytokine profile to the Th2 profile [1]. Certain Th1 predominant autoimmune diseases such as rheumatoid arthritis tend to show improvement during pregnancy, while Th2 predominant diseases such as systemic lupus erythematosus tend to flare [2]. Neonatal vasculitis in infants born to mothers with systemic lupus erythematosus, Sjögren’s syndrome and systemic vasculitides have been described. Here we report the first case, to our knowledge, of a new onset of seronegative inflammatory arthritis in a primiparous mother associated with the development of an effectively treated systemic vasculitis syndrome in the neonate.

### Clinical case

A Caucasian male infant was born at 33 weeks and 4 days gestation via caesarian section, due to fetal tachycardia, following spontaneous premature rupture of membranes. APGAR scores were 9 at 1 min and 9 at 5 min. Birth weight was 2920 g (90th percentile), head circumference 34.5 cm (>97th percentile), length of 44 cm (40th percentile). There were no dysmorphic features. Within the first 24 h of life, blue discoloration was noted to the distal left second finger which progressed to multiple fingers and toes in all four limbs. The infant was transferred to a tertiary care neonatal intensive care unit for evaluation.

The mother, a 28-year-old Caucasian woman, developed new onset symmetrical polyarthritis during her first trimester of pregnancy. Past history was significant for a previous miscarriage at 5 weeks gestation and a cholecystectomy. Family history was negative. There was no smoking, alcohol or drug use during pregnancy. Prenatal screening showed rubella titre indeterminant (7.4 IU/ml) as well as hepatitis B, human immunodeficiency virus, syphilis negative. Parvovirus B19 immunoglobulin G (IgG) reactive, cytomegalovirus IgG non-reactive, toxoplasmosis IgG negative.

At 6 weeks’ gestation, the mother underwent dental work complicated by an oral abscess requiring 7 days of amoxicillin. Two days after initiation of amoxicillin, she developed severe myalgias, episodes of nausea, vomiting and diarrhea which resolved in a few days. There was no associated fever, rash, or urinary symptoms. At 8 weeks gestation she presented with severe polyarthritis affecting large and small joints with significant functional impairment. She was prescribed oral prednisone (15 mg oral daily) at 10 weeks’ gestation for a presumed reactive arthritis. Blood work prior to treatment showed elevated inflammatory markers including C-reactive protein (CRP) of 210 mg/L (normal < 3 mg/L) and erythrocyte sedimentation rate (ESR) of 60 mm/hr. (normal 0-20 mm/hr). She was steroid dependent for the remainder of the pregnancy requiring high dose prednisone (25 mg orally twice daily) with return of symptoms below this dose.

Additional infectious work up was complete including Epstein Barr virus and Lyme serology which were negative. Prior to treatment her anti-nuclear antibody (ANA), extractable nuclear antigen antibodies (ENA) including anti-Ro and anti-La, anti-double stranded DNA (anti-dsDNA), antiphospholipid antibodies (APLA), antineutrophilic cytoplasmic antibodies (ANCA), rheumatoid factor (RF), anti-cyclic citrullinated peptide (anti-CCP), complement levels (C3, C4) were negative or within normal range.

**Fig. 1:**
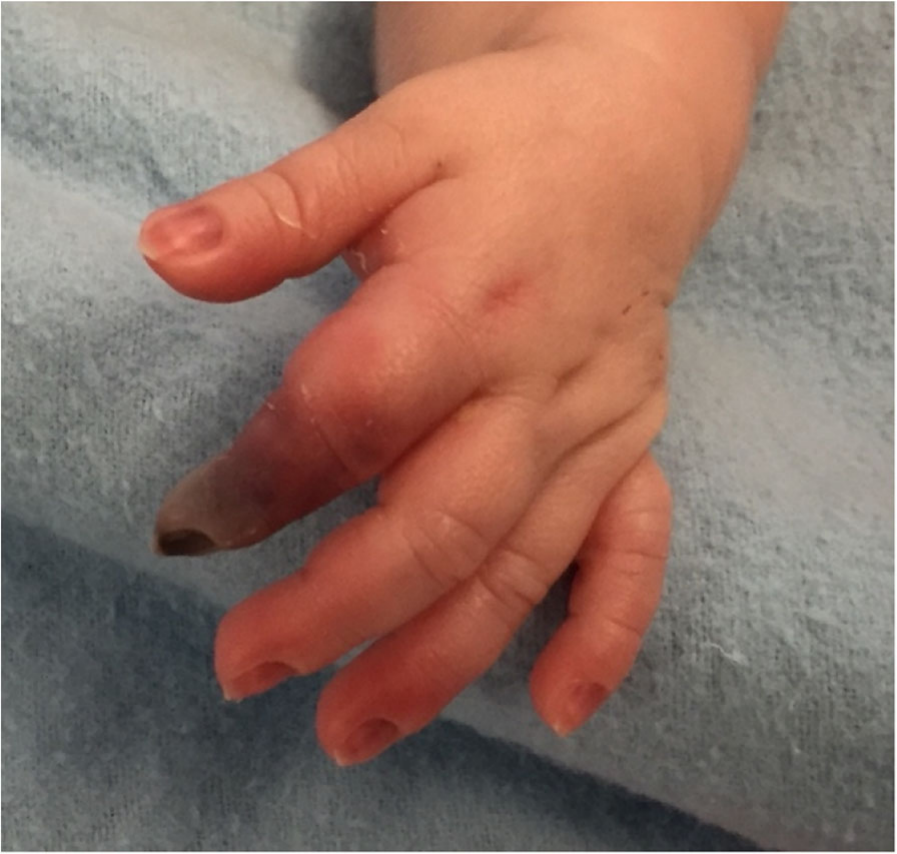
Left hand on fifth day of life

The neonate was transferred to a tertiary care neonatal intensive care unit (NICU) on day 3 of life and had a negative full septic work up including blood, urine and cerebrospinal fluid cultures. Despite treatment with antibiotics (Vancomycin and Cefotaxime), fresh frozen plasma (received every 12 h for 7 days) and topical nitroglycerin, the discoloration remained, particularly in the left hand (Fig. 1). Stress dosing of hydrocortisone for possible adrenal insufficiency (no cortisol level performed) was given due to the long in-utero exposure to steroids in the mother. Heparin was given for potential inherited thrombophilia and transitioned to Enoxaparin. On day 11 of life, he was noted to have erythema of his hands and feet, which prompted initial consideration for neonatal Kawasaki disease (no other clinical criteria for Kawasaki disease and he was afebrile at the time of first intravenous immunoglobulin (IVIG) administration). After two doses of IVIG (2 g/kg) short lived improvement was noted. Electrocardiogram (ECG) was normal with no signs of dysrhythmia. An echocardiogram showed enlarged coronary arteries (maximum in left anterior descending artery (LAD), z-score 3.93) with normal anatomy and systolic function. Two days after the second dose of IVIG, fever returned (up to 38.2 degrees Celsius), he had tachycardia, elevated CRP (maximum CRP of 172 mg/L, normal < 3 mg/L) and oral prednisolone (2 mg/kg divided twice daily) was initiated. Inflammatory markers, skin discoloration and tachycardia improved with steroid treatment. Repeat echocardiogram 1 week after starting prednisolone demonstrated normal coronaries.

**Fig. 2:**
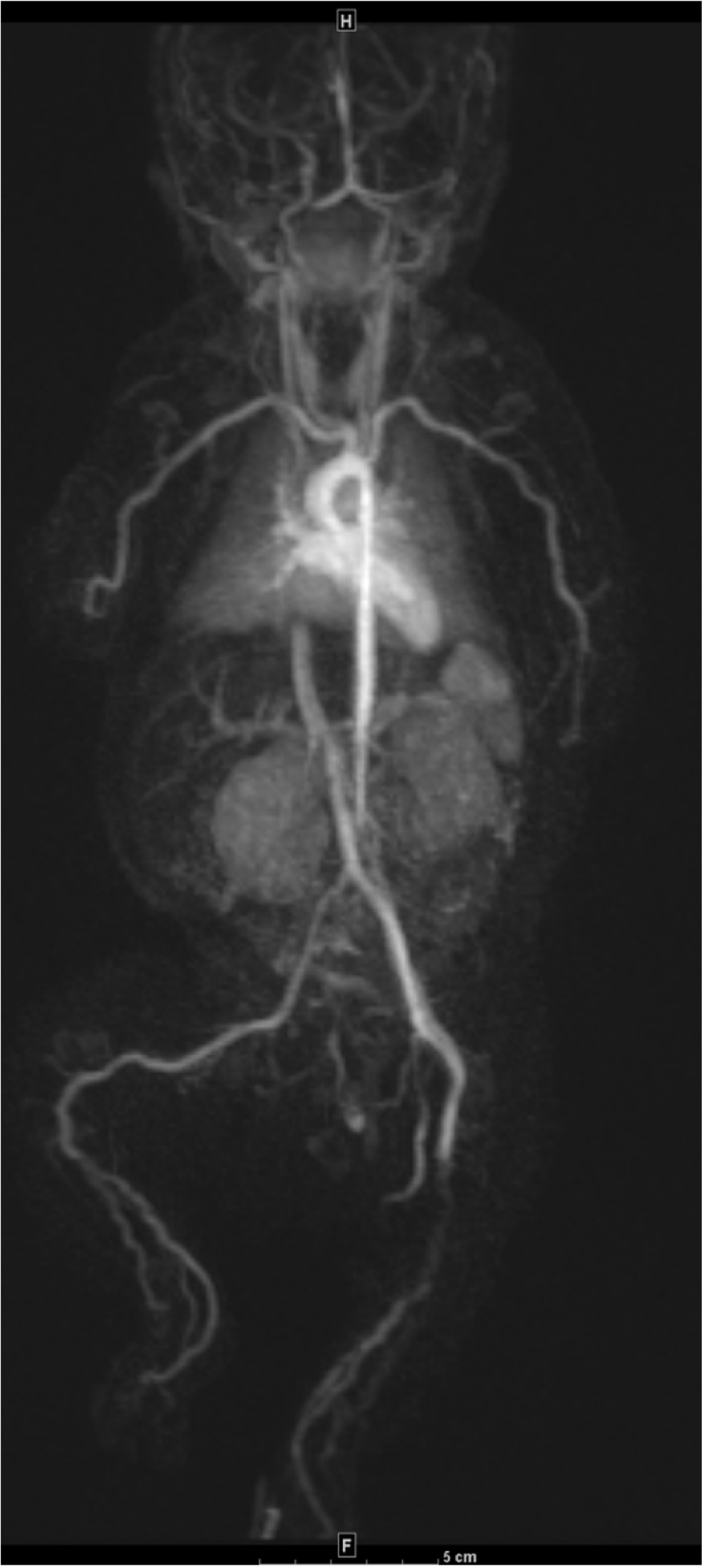
Magnetic resonance angiography at one month of age. Gadolinium enhancing irregular and tortuous appearing medium sized arteries bilaterally in the upper and lower extremities

Full body magnetic resonance angiography (MRA) was significant for abnormalities in the upper limbs with tortuosity and gadolinium enhancement of the brachial as well as the distal axillary arteries. In the lower limbs, tortuosities and gadolinium enhancement involving distal femoral, popliteal, posterior and anterior tibialis arteries suggestive of vasculitis or vasculopathy (Fig. 2). Aortic, pulmonary, renal and celiac trunk vessels were normal. Given the clinical syndrome, imaging findings and response to corticosteroid administration, vasculitis was favored.

The neonate’s autoimmune work included a negative ANA, ENA (including anti-Ro/La), APLA, ANCA, RF, and anti-smooth muscle antibody (ASMA). Complement levels (C3, C4, CH50) and immunoglobulins (IgG, IgA, IgM) were normal. Thrombophilia workup including INR, PTT, fibrinogen, D-dimer, protein C, protein S, antithrombin, homocysteine level, Factor VIII level, lupus anticoagulant, prothrombin mutation were within normal limits or negative. Work up for neonatal lupus erythematosus showed a normal ECG and echocardiogram with no signs of cardiomyopathy or endocardial fibroelastosis. Ophthalmology exam was normal, with no signs of retinal vasculitis. Newborn screen (Additional file 1) was negative and his metabolic work up including ammonia level, plasma amino acids, urine organic acids, total and free carnitine, acylcarnitine profile, methylmalonic acid level were normal. His DNA was screened with targeted sequencing for known hereditary autoinflammatory diseases (17 next-generation sequencing panel including Deficiency of Adenosine Deaminase 2 (DADA2) and STING-associated vasculopathy with infantile onset (SAVI)), however, we were not able to identify a causative mutation. Whole exome sequencing was conducted as a trio (both Caucasian parents and the affected child were sequenced simultaneously), which did not identify a pathogenic variant.

**Fig. 3:**
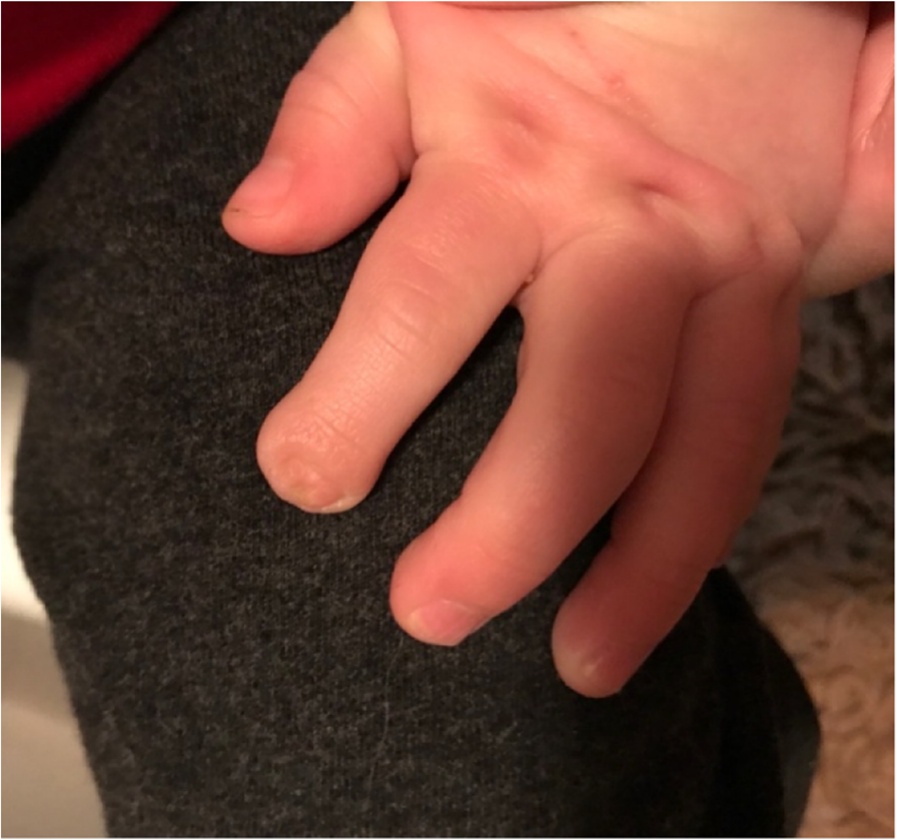
Left hand at 21 months of age

**Fig. 4:**
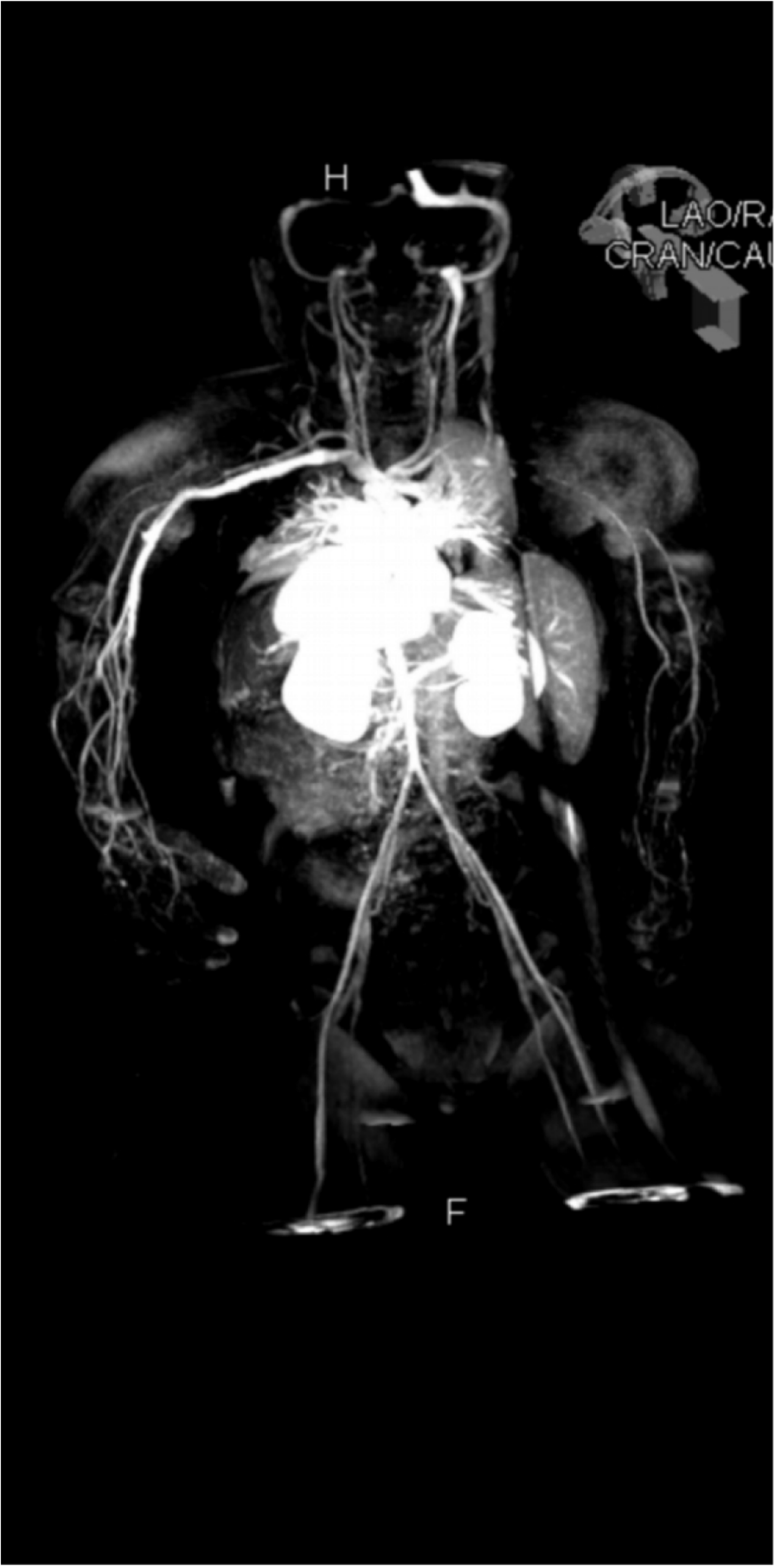
Follow-up magnetic resonance angiography at 17 months of age demonstrates residual tortuosity of forearm vessels with normalization of lower extremity abnormalities and no gadolinium enhancement

Oral prednisolone was continued with improvement of the discoloration in the infant’s hands and feet, however the left index finger discoloration persisted with progression to necrosis. A diagnostic biopsy was considered but was deferred due to the necrosis and likely low diagnostic yield. Enoxaparin was discontinued at 3 months of age. The male infant was effectively weaned off steroids by 5 months of age, he is now 4 years old with normal growth and development. A follow up image of the hand at 21 months old is shown in Fig. 3. Repeat MRA at 17 months of age showed residual mild tortuosity of the upper extremity abnormalities with no gadolinium enhancement and the lower limbs arteries normal in appearance with normalization of prior tortuosities (Fig. 4).

The mother was seen by an adult rheumatologist post-partum and repeat autoantibody profile remained negative (ANA, RF and anti-CCP). She was weaned off prednisone with a slow taper of approximately 3 months and was treated with hydroxychloroquine for a total of 8 months. She remains in remission off therapy and has had no further pregnancies.

## Discussion

Vasculitides are rarely seen in neonates, with the exception of reported vasculitis occurring in infants of mothers affected by systemic lupus erythematosus, Sjögren’s syndrome, and systemic vasculitides including Behçet’s disease, polyarteritis nodosa as well as microscopic polyangiitis (MPA).

Stone et al. describes a pregnant 24-year-old Caucasian woman with a history of cutaneous polyarteritis nodosa (PAN) who had a flare during pregnancy and treated with prednisone [3]. The newborn infant born at 36 weeks gestation had a “dusky” episode on day 1 of life, then developed cyanosis of her left finger, left ear, nose, upper lip and several toes. She had prominent livedo reticularis on the proximal extremities and abdomen with development of dermal nodules on the trunk, extremities as well as the scalp. She was started on heparin and prednisone with improvement. Similar to our case, cyanosis improved but the infant suffered from autoamputation of parts of three toe digits. A skin nodule biopsy showed results consistent with medium sized vasculitis suggestive of polyarteritis nodosum [3]. Both mother and baby were ANA, RF and ANCA negative. The authors propose the presentation was caused by a maternal factor crossing the placenta. In addition, the neonate was found to have high levels of fibrinogen, however low functioning, which could be secondary to an abnormal fibrinogen molecule or antibody interfering with functioning which could lead to a thrombophilia. The neonate we present had fibrinogen levels within normal range (2.86–3.37 g/L).

The mother, in our case, presented at 8 weeks gestational age with polyarthritis requiring high dose steroids. Angiogenesis and vasculogenesis begins at approximately week 3 of pregnancy. Blood vessels continue to form in the embryo and placenta until approximately 10 weeks, when most organs are completely formed [4]. Our patient was extensively investigated for infectious, thrombophilia and genetic etiologies for a vasculitis syndrome which were negative. Given his prematurity, there is further reduction in function of the Vitamin K dependent coagulation factors and contact factors making thrombosis causing finger necrosis very unlikely [5, 6].

Bansal et al. described a case of a 32-year-old woman with a history of polyarteritis nodosa (PAN), anti-myeloperoxidase (MPO) positive and delivery of a premature female, by emergency caesarean section for pre-eclampsia [7]. The newborn developed pulmonary hemorrhage requiring ventilatory support in addition to proteinuria and hematuria. Treatment included hydrocortisone (3 mg/kg) and plasma exchange which led to improvement. Umbilical cord blood showed elevated anti-MPO antibody at 3.30 U/mL (reference range 0.00–0.89 U/mL) which normalized with therapy. Hydrocortisone was successfully tapered over 3 weeks. This case presenting with a mother with a known history of PAN causing a transient vasculitis syndrome in her newborn presumed related to placentally transferred autoantibody. Silva et al. described a 41-year-old woman with a history of MPA, anti-MPO and anti-glomerular basement membrane positive who delivered a healthy term baby boy [8]. Her disease remained in remission during pregnancy with continued maintenance immunosuppression including low dose prednisone (7.5 mg/day) and azathioprine (125 mg/day). Despite high levels of anti-MPO (greater than 100 U/mL) measured in the newborn immediately after birth, he did not develop any signs of vasculitis. This suggests that the presence of anti-MPO antibody positivity alone is not pathogenic and may require other factors or an inflammatory stimulus [8]. The rarity of systemic vasculitis in neonates from known vasculitis in their mothers may then be explained by a combination of factors as well as disease control during pregnancy.

During pregnancy, the hormonal profile causes immunomodulatory changes, with a shift from a Th1 to a Th2 lymphocyte predominant response [1]. Newer research has suggested it may be a combination of other T cells and inflammatory markers including Th17, T regulatory cells, tumor necrosis factor as well as IL-2 allowing tolerance of the fetus that allows this [9]. Typically, rheumatoid arthritis improves in pregnancy (Th1 predominant disease) while certain diseases such as systemic lupus erythematosus (Th2 predominant disease) tend to flare [2]. The presence of IgG isotype autoantibodies in maternal diseases can cross the placenta causing antibody-mediated damage in the neonate [10]. This includes neonatal lupus erythematosus (NLE) seen in mothers with anti-Ro/anti-La antibodies passing through the placenta, immune thrombocytopenic purpura and antiphospholipid syndrome. Clearance of these passively transferred maternal antibodies occurs by 6 months of age. In our case, the timing of prednisolone wean was concurrent with expected timing for clearance of a suspected maternal antibody or factor. At 5 months of age, steroids were successfully discontinued with no return of symptoms.

## Conclusion

This is a unique case of new onset seronegative presumed reactive arthritis in a mother with the rare development of a successfully treated medium vessel vasculitis in an infant. The etiology for the infant’s symptoms is unknown but may be secondary to a transplacental transfer of an unidentified autoantibody, protein, cytokine or a combination of these factors with an inflammatory stimulus which may explain his presentation. This theory is supported with complete recovery following treatment in the neonate by 6 months of age which coincides with timing for clearance of maternal factors.

## Supplementary Information

**Additional file 1.**

## Data Availability

Patient data used during this report are available from corresponding author on reasonable request.

## Abbreviations

ANA: Anti-nuclear antibody
ANCA: Antineutrophilic cytoplasmic antibodies
APGAR: Appearance, pulse, grimace, activity and respiration
APLA: Antiphospholipid antibodies
ASMA: Anti-smooth muscle antibody
CRP: C-reactive protein
DADA2: Deficiency of Adenosine Deaminase 2.
ECG: Electrocardiogram
ENA: Extractable nuclear antigen antibodies
ESR: Erythrocyte sedimentation rate
INR: International normalized ratio
IVIG: Intravenous immunoglobulin
LAD: Left anterior descending
MPA: Microscopic polyangiitis
MPO: Anti-myeloperoxidase
MRA: Magnetic resonance angiography
NICU: Neonatal intensive care unit
NLE: Neonatal lupus erythematosus
PAN: Polyarteritis nodosa
PTT: Partial thromboplastin time
RF: Rheumatoid factor
SAVI: STING-associated vasculopathy with infantile onset
