# Supplementary material for: A case report of a severe neonatal systemic vasculitis on the first day of life
Source: Pediatr Rheumatol Online J. 2021 Oct 30;19:154. doi: 10.1186/s12969-021-00618-x (PMC8556936; doi:10.1186/s12969-021-00618-x)
Supplement: Supplementary file 1 — Additional file 1. [file 12969_2021_618_MOESM1_ESM.docx]

**Appendix 1.**

Newborn screening Ontario (https://www.newbornscreening.on.ca/en)

**Complete list**:

Argininosuccinic Acid Lyase Deficiency (ASA)

Biotinidase Deficiency

Carnitine Uptake Defect (CUD)

Citrullinemia

Cobalamin A & B Defects

Congenital Adrenal Hyperplasia (CAH)

Congenital Hypothyroidism (CH)

Critical Congenital Heart Disease (CCHD)

Cystic Fibrosis (CF)

Galactosemia

Glutaric Acidemia Type 1 (GA1)

Homocystinuria

Hurler Disease (“Mucopolysaccharidosis type 1H” or “MPS1H”)

Isovaleric Acidemia (IVA)

Long Chain 3-Hydroxyacyl-CoA Dehydrogenase Deficiency (LCHAD)

Maple Syrup Urine Disease (MSUD)

Medium Chain Acyl CoA Dehydrogenase Deficiency (MCADD)

Methylmalonic Acidemia (MMA)

Phenylketonuria (PKU)

Propionic Acidemia (PA)

Severe Combined Immune Deficiency (SCID)

Sickle Cell Disease (Hemoglobin SC)

Sickle Cell Disease (Hemoglobin SS)

Sickle Cell Disease (Sickle/Beta-Thalassemia)

Spinal Muscular Atrophy (SMA)

Trifunctional Protein Deficiency (TFP)

Tyrosinemia Type 1

Very Long Chain Acyl CoA Dehydrogenase Deficiency (VLCAD)
